# Mitochondrial Sensorineural Hearing Loss: A Retrospective Study and a Description of Cochlear Implantation in a MELAS Patient

**DOI:** 10.1155/2012/287432

**Published:** 2012-02-20

**Authors:** Mauro Scarpelli, Francesca Zappini, Massimiliano Filosto, Anna Russignan, Paola Tonin, Giuliano Tomelleri

**Affiliations:** ^1^Division of Clinical Neurology, Department of Neurological, Neuropsychological, Morphological and Movement Sciences, University of Verona, 37134 Verona, Italy; ^2^Division of Clinical Neurology, Section for Neuromuscular Diseases and Neuropathies, University Hospital “Spedali Civili”, 25123 Brescia, Italy

## Abstract

Hearing impairment is common in patients with mitochondrial disorders, affecting over half of all cases at some time in the course of the disease. In some patients, deafness is only part of a multisystem disorder. By contrast, there are also a number of “pure” mitochondrial deafness disorders, the most common probably being maternally inherited. We retrospectively analyzed the last 60 genetically confirmed mitochondrial disorders diagnosed in our Department: 28 had bilateral sensorineural hearing loss, whereas 32 didn't present ear's abnormalities, without difference about sex and age of onset between each single group of diseases. We reported also a case of MELAS patient with sensorineural hearing loss, in which cochlear implantation greatly contributed to the patient's quality of life. Our study suggests that sensorineural hearing loss is an important feature in mitochondrial disorders and indicated that cochlear implantation can be recommended for patients with MELAS syndrome and others mitochondrial disorders.

## 1. Introduction

Mitochondrial diseases are disorders caused by impairment of the mitochondrial respiratory chain. The genetic error can affect both mitochondrial DNA (mtDNA) and nuclear DNA (nDNA) [1].

MtDNA mutations are classified as either large-scale rearrangements (partial deletions or duplications), usually sporadic, or point mutations, which are usually maternally inherited, and concern genes responsible for protein synthesis (rRNAs or tRNAs), or genes encoding subunits of the electron transport chain (ETC) [2]. The phenotypic expression of mtDNA mutations depends on the affected gene, its tissue distribution, and the different dependency of different organs and tissues on the mitochondrial energy supply. Visual and auditory pathways, heart, central nervous system (CNS), and skeletal muscle are the tissues mostly involved, because of their dependence on aerobic energy production [1].

Hearing impairment is common in patients with mitochondrial disorders, affecting over half of all cases at some time in the course of the disease [3]. Although the final common pathway for the hearing loss is thought to involve ATP deficiency secondary to a biochemical defect of the respiratory chain, the clinical presentation of mitochondrial deafness varies considerably, both in terms of associated clinical features and of natural history. In some patients, deafness is only part of a multisystem disorder, often involving the central nervous system, neuromuscular system, or endocrine organs; in other cases, deafness may represent a feature of an oligosyndromic disease [4].

By contrast, there are also a number of mitochondrial “pure” deafness disorders, the most common probably being maternally inherited deafness due to the A1555G mutation in the 12 s rRNA gene, MTRNR1 [5]. The use of streptomycin and to a lesser extent other aminoglycoside antibiotics can cause hearing loss in genetically susceptible individuals. These drugs are known to exert their antibacterial effects at the level of the decoding site of the small ribosomal subunit, causing miscoding or premature termination of protein synthesis [6]. The hearing loss is primarily high frequency and may be unilateral. Risk factors for aminoglycoside ototoxicity include therapy lasting more than 7 days, elevated serum levels, prior exposure to aminoglycosides, noise exposure, high daily dose, use in neonates, and a background of predisposing mutations. Several mutations in the MTRNR1 gene encoding the 12S rRNA (961delT/insC, T1095C, C1494T, A1555G, and possibly A827G, T1005C and A1116G) and possibly also mutations (G7444A) in the COI/MTTS1 gene overlap can contribute to ototoxic hearing loss [7–11]. The MTRNR1 mutations probably alter the secondary structure of the 12S rRNA molecule, so that it resembles its bacterial counterpart, the 16S rRNA, more closely. As the bacterial 16S rRNA molecule is the target of aminoglycoside action, this might explain the cumulating effect of these MTRNR1 mutations and the use of aminoglycosides [12]. Mitochondrial nonsyndromic sensory neural hearing loss (SNHL) is also associated with the A7445G, 7472insC, T7510C, and T7511C mutations in the tRNASer (UCN) gene, MTTS1 [13].

The pathological examination of the inner ear is technically demanding, highly specialized, and only possible postmortem. There have, therefore, only been a few detailed pathological studies of the auditory system in patients with mitochondrial diseases. In the cochlea, the stria vascularis maintains the ionic gradient necessary for sound transduction and the complex interaction between inner and outer hair cells [14]. These components are highly metabolically active, and it is likely that a respiratory chain defect and the attendant relative deficiency of intracellular ATP would impair the function of both the stria and the air cells, ultimately leading to cell death, possibly through apoptosis [15].

Hearing loss is usually peripheral (due to cochlear or auditory nerve dysfunction), but in patients with a multisystem mitochondrial disorder, the auditory system may be affected at the brain stem, midbrain or at a higher level in the auditory cortex. The peripheral hearing loss typically affects high frequencies first, followed by intermediate frequencies, and finally involving low frequencies and causing the typical “flat” audiogram seen in a severely deaf individual. The preferential involvement of high frequencies may be related to the relatively high energy requirements of the basal cochlea [16]. The vast majority of patients with mitochondrial deafness have absent otoacoustic emission, providing strong evidence that the cochlea is the component most sensitive to mitochondrial dysfunction [17, 18].

In this review, we analyzed the results of a retrospective study about the presence of hearing loss in a cohort of patients with genetically confirmed mitochondrial disease and we described the results and follow-up of a MELAS patient who underwent cochlear implantation.

## 2. Patients and Methods

### 2.1. Patients

We retrospectively analyzed the last 60 genetically confirmed mitochondrial disorders diagnosed in our Department in order to identify patients affected with hearing loss. Males and females were quite equally distributed (33 males and 27 females), with an age at diagnosis ranging from 8 to 73 years; the mean age of this group of patients was 45 years old. Three patients belonged to the same family.

### 2.2. Methods

Most of patients were referred to our centre for a suspected mitochondrial disease and underwent a muscle biopsy (quadriceps or deltoid muscle). In these cases, DNA was extracted from frozen muscle tissue and used for direct sequencing of mitochondrial and/or nuclear genes, with standard methods [19, 20]. In some other cases, family history was strongly suggestive for mitochondrial disorder so that patients were not submitted to muscle biopsy, and DNA to perform molecular analysis was extracted from their blood.

Presence of single or multiple deletions of mitochondrial DNA was revealed by long-range PCR or southern blot analysis, using standard methods [20]. *mtDNA* point mutations were searched by screening the whole *mtDNA* using the MitoScreen Assay Kit for the Transgenomic WAVE System and DHPLC (denaturing high-performance liquid chromatography) with single-stranded conformational polymorphism analysis, following supplier's indications or by using the ABI PRISM BigDye Terminators v3.0 Cycle Sequencing Kit.

All of the cases included in this study performed audiometric examination before to receive the genetic diagnosis. The cut-off of hearing loss was defined according to the mean hearing loss at frequencies of 250, 500, 1000, 2000, and 4000 Hz as follows: normal hearing ≤ 20 dB hearing loss.

## 3. Results

**Figure 1:**
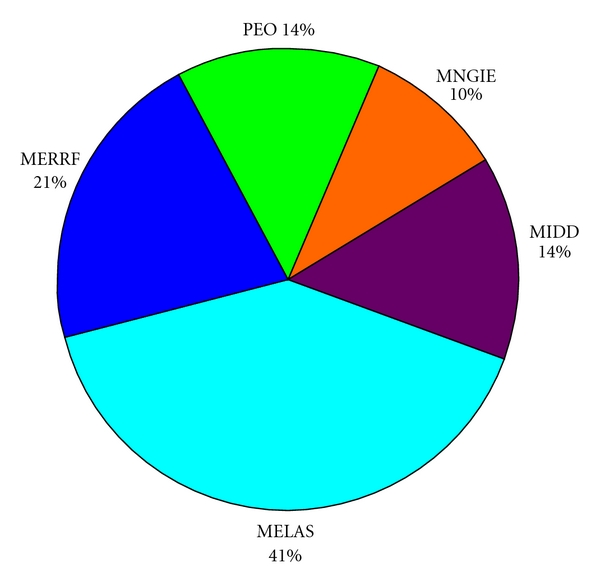
Graph of distribution of hearing loss into the clinical syndromes.

Out of the 60 cases, 28 had bilateral sensorineural hearing loss, whereas 32 did not present ear's abnormalities. Clinical findings and the frequency of SNHL are summarized in Table 1 and Figure 1. All of the three patients belonging to the same family had A3243G mitochondrial DNA mutation and hearing loss.

One of the 28 patients with hearing loss, a MELAS patient, was submitted to cochlear implantation. Following, we briefly reported his main clinical notes.

He was a 46-year-old man who started to complain mild bilateral hearing loss from his 20; he had normal prenatal and perinatal histories. His family history was also normal. Diabetes mellitus was noted at the age of 33, and insulin therapy was initiated. Serum biochemical studies showed a high level of lactic acidosis, which increased after exercise, and echocardiography disclosed the presence of hypertrophic cardiomyopathy. Neurological examination showed diffuse mild muscle weakness, more evident in distal lower limbs, with stepping gait. Computer tomography (CT) and magnetic resonance imaging (MRI) scans showed no abnormality in either inner ear. MRI demonstrated mild cerebellar atrophy.

Muscle biopsy revealed strong reactive vessels on Succinate Dehydrogenase stain (SDH), without ragged red neither citochrome C-oxidase negative fibers. Molecular analysis revealed the presence of the A3243G mitochondrial DNA heteroplasmic mutation.

During next years, hearing loss markedly worsened: pure-tone audiometry revealed bilateral profound hearing loss with an average of 130 dB in both ears. The hearing aid was no longer useful. In a promontory stimulation test, the patient responded to electrical stimulation in both ears at the intensity of 0.3 mA.

At 33 years old, he underwent cochlear implantation surgery using a 24-channel device (cochlear-contour) in the right ear. Twenty-five electrode rings were successfully inserted and all 22 channels and 2 extracochlear electrodes were found to be usable at the initial “switch on” of the cochlear implant.

Clinical followup was quite stable: he referred sometimes episodes of gastrointestinal pseudoobstruction and few periods of scarce glycemic control; outcome of cochlear implantation was good: postoperative neural response telemetry (NRT), implant-evoked BAEP and middle latency response (MLR) showed good responses. Eight years after the surgery, the patient could use the telephone and was satisfied with the improvement in communication due to the cochlear implant.

## 4. Discussion

Hearing impairment is a common feature of mitochondrial disease, either in isolation, or as a part of a complex multisystem disorder, with the cochlea bearing the brunt of the pathology. Ultimately, all forms of mitochondrial deafness arise through a respiratory chain defect causing ATP depletion, but it is not clear why hearing should be preferentially affected in some mitochondrial disorders and not in others, nor why cochlear pathology can vary between different disorders. There is clear evidence of a major environmental influence in some forms of mitochondrial deafness, and the interaction between nuclear and mitochondrial genes appears to be important [21].

The degree of hearing loss correlates well with the mutation load in skeletal muscle [3], and the progressive nature of the hearing loss may be related to the accumulation of mutated mtDNA within the cochlea. Although this appears to be the general trend, there are clear exceptions to the rule. In one patient with the A3243G mutation, severe hearing loss was associated with low levels of mutated mtDNA in skeletal muscle [22]. This may occur because of unequal segregation of mutated mtDNA among different tissues during early development, so that occasionally, by chance, high levels are present in the cochlear precursors, and lower levels in skeletal muscle precursor cells.

The percentage of mutated mtDNA undoubtedly contributes to the clinical variability seen among patients, but this does not provide the whole explanation. It is not currently known why certain maternal pedigrees transmitting A3243G tend to develop a pure deafness-diabetes phenotype, whereas others only show ptosis and external ophthalmoplegia, and yet others are affected by severe multisystem MELAS phenotype [23]. Additional genetic factors are likely to be important, but have yet to be identifies [21].

Our study considers only the syndromic form of mitochondrial SNHL, showing that the frequency of hearing loss in our group of patients is the same of the most of studies reported in literature [3, 21]; moreover, it suggests how this form does not present difference about sex and age of onset into each single group of diseases (data not shown). One limit of our study is the retrospective analysis that does not allow to define in patients without hearing loss if this deficit will develop in the future and does not give the real severity of hearing loss within different categories.

However, the study offers important considerations, like the relative low frequency of hearing loss in patients with chronic progressive external ophthalmoplegia (CPEO) (16%), which could be considered in most of cases a “pure” myopathy, and, by contrast, the high frequency of hearing loss in patients affected by mitochondrial neurogastrointestinal encephalomyopathy (MNGIE) (75%), in which the diagnosis may be difficult at the beginning and the presence of hearing loss could orient clinicians in considering a mitochondrial disease in the differential diagnosis [24].

The case presented in this study remarks the importance to consider cochlear implant in patients with mitochondrial SNHL. The patient suffered from hearing loss due to MELAS syndrome; after cochlear implantation his quality of life markedly improved and he could preserve his work.

Since the first recorded cochlear implant in a patient with Kearns-Sayre syndrome [25], many patients have successfully received implants [13]. In many ways, patients with mitochondrial disease are “ideal” recipients of a cochlear implant because the hearing loss develops well after speech development, and often in isolation (as in patients with diabetes and deafness due to A3243G, or non-syndromic deafness due to A1555G). A systematic review of literature (March 2003) identified 12 detailed descriptions of patients with mitochondrial sensorineural deafness who had cochlear implants [13]. All 12 cases had profound postlingual deafness. The age of onset of the deafness and the age at surgery varied, but 58% were able to converse on the telephone following the procedure, and the remainder had good open-set speech recognition. There were no reported complications. The procedure should, however, only be undertaken with caution, because the implantation procedure requires a general anesthetic and takes a number of hours, and because it is also important to consider the natural history of the disorder in the individual patient. This is the case of MNGIE, in which the mean age of death is 37 years [26], but the presence of new therapeutic options gives hopes for patients with this devastating neurodegenerative disorder and could modify the prognosis. Very recently, Li and colleagues described a successful multichannel cochlear implantation in a 28-years-old MNGIE woman [27], confirming the importance to consider and treat ear's problems also in these types of disorders.

In conclusion, individuals who harbour mtDNA mutations may be at risk of developing severe hearing deficit, and these individuals should avoid ototoxic agents, such as aminoglycoside antibiotics, which may further compromise cochlear function [3].

Our study suggests that SNHL is an important feature in mitochondrial disorders and should be considered in the diagnostic workup and management of patients with suspected mitochondrial disease. We found that cochlear implantation in a patient with multisystem degenerative disease greatly contributed to the patient's quality of life and made it possible for him to communicate with family members and caregivers better than previously.

The results indicated that cochlear implantation can be recommended for patients with MELAS syndrome and other mitochondrial disorders, if they have residual retrocochlear function.

## Figures and Tables

**Table 1:** Clinical and molecular features of patients analyzed in our study.

Clinical syndrome	Molecular defect	Hearing loss	Non hearing loss	Total
MIDD	A3243G	4	0	4
MELAS	A3243G	11	10	21
MERRF	A8344G	6	1	7
PEO	Single deletion (4) Multiple deletions (20)	4	20	24
MNGIE	TYMP mutations	3	1	4

MIDD: Mitochondrial inherited ciabetes and deafness; MELAS: mitochondrial encephalomyopathy with lactic acidosis and stroke-like episodes; MERRF: myoclonic epilepsy with ragged red fibers; PEO: progressive external opthalmoplegia; MNGIE: mitochondrial neurogastrointestinal Encephalomyopathy.
